# Supplementary material for: Multiplexed RNA profiling by regenerative catalysis enables blood-based subtyping of brain tumors
Source: Nat Commun. 2023 Jul 17;14:4278. doi: 10.1038/s41467-023-39844-0 (PMC10352249; doi:10.1038/s41467-023-39844-0)
Supplement: Supplementary file 1 — Supplementary Information [file 41467_2023_39844_MOESM1_ESM.pdf]

**SUPPLEMENTARY INFORMATION**

# Multiplexed RNA profiling by regenerative catalysis enables blood-based subtyping of brain tumors

Yan Zhang[1,2,#], Chi Yan Wong[1,3,#], Carine Z.J. Lim[1,2,#], Qingchang Chen[1,2], Zhonglang Yu[1,2], Auginia Natalia[1,2], Zhigang Wang[1], Qing You Pang[4], See Wee Lim[4], Tze Ping Loh[1,5], Beng Ti Ang[6,7], Carol Tang[4,7,8], Huilin Shao[1,2,9,10,11,*]

[1] Institute for Health Innovation & Technology, National University of Singapore, Singapore
[2] Department of Biomedical Engineering, College of Design and Engineering, National University of Singapore, Singapore
[3] Department of Medicine, Yong Loo Lin School of Medicine, National University of Singapore, Singapore
[4] Neuro-Oncology Research Laboratory, Department of Research, National Neuroscience Institute, Singapore
[5] Department of Laboratory Medicine, National University Hospital, Singapore
[6] Department of Neurosurgery, National Neuroscience Institute, Singapore
[7] Duke-National University of Singapore Medical School, Singapore
[8] School of Biological Sciences, Nanyang Technological University Singapore, Singapore
[9] National Neuroscience Institute, Singapore
[10] Department of Surgery, Yong Loo Lin School of Medicine, National University of Singapore, Singapore
[11] Institute of Molecular and Cell Biology, Agency for Science, Technology and Research, Singapore
[#] These authors contributed equally

* Corresponding author
Huilin Shao, PhD
National University of Singapore
MD6, 14 Medical Drive
#14-01, Singapore 117599
(65) 6601 5885
huilin.shao@nus.edu.sg

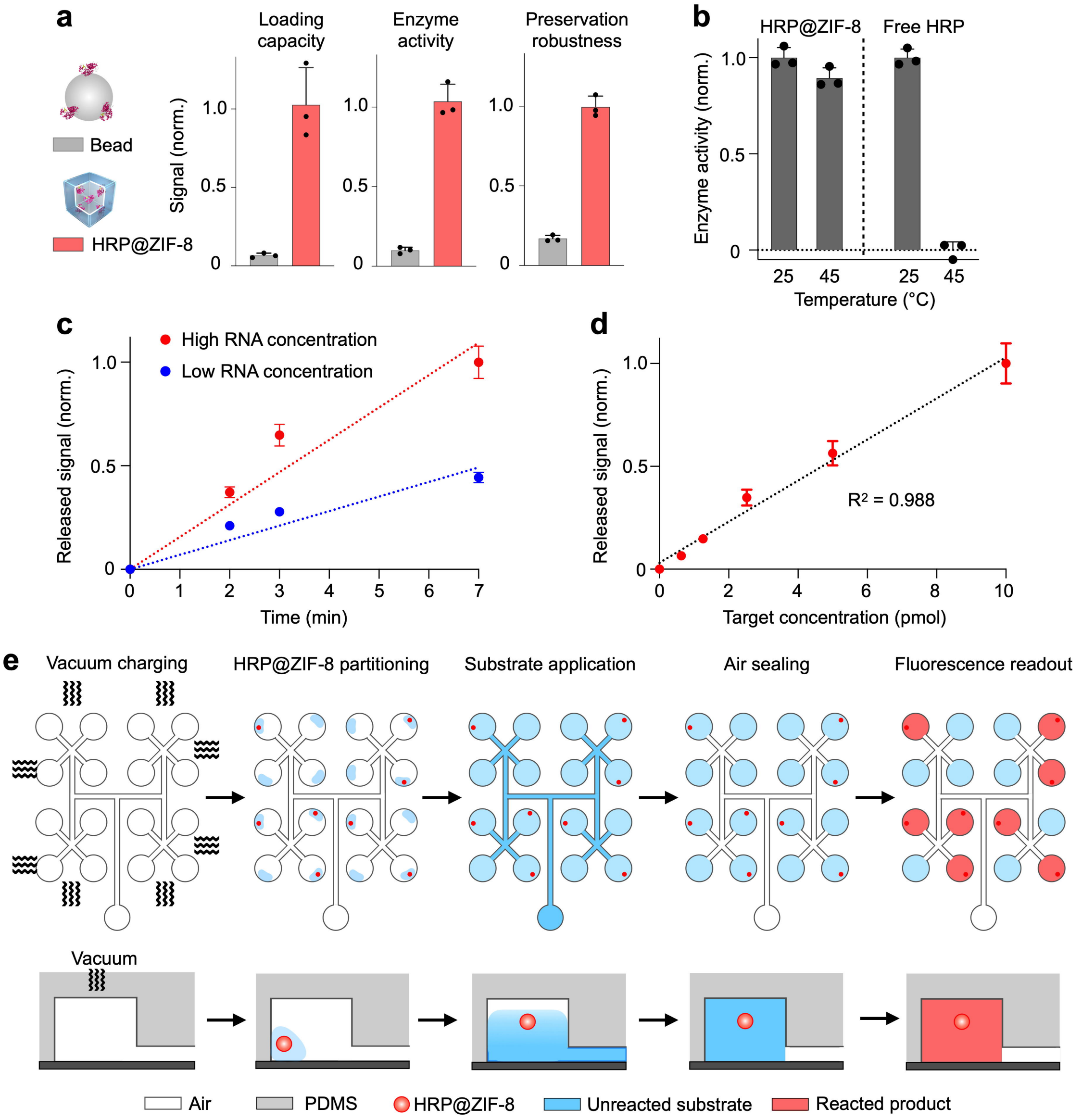


**Supplementary Figure 1. EZ-READ for catalytic digital quantification of RNAs.**
**(a)** Enhanced performance of HRP@ZIF-8. As compared to HRP-functionalized polystyrene beads bearing equal amounts of the protein enzyme, HRP@ZIF-8 showed more efficient catalysis, with a higher loading capacity and improved enzyme activity. When subjected to lyophilization treatment, HRP@ZIF-8 showed more robust activity recovery upon rehydration. **(b)** Temperature stability of HRP@ZIF-8. As compared to free HRP, HRP@ZIF-8 could withstand high temperature, and showed negligible loss of enzyme activity. **(c)** Regenerative transduction. Using FITC-labled HRP@ZIF-8 nanoparticles, we measured their release from the transducers over time when incubated with different concentrations of RNA targets (high: 10 pmol and low: 3 pmol). Fluorescence signal was measured from the supernatants. EZ-READ showed continuous nanoparticle release, reflective of the input RNA amounts. **(d)** Correlation to RNA target concentration. The number of released HRP@ZIF-8 nanoparticles showed a linear relationship with target concentration. **(e)** Schematics of catalytic digital quantification. We perform a series of operations, namely vacuum charging, HRP@ZIF-8 partitioning, substrate application, air sealing and fluorescence readout. The sample RNA concentration can be determined by counting the number of fluorescent microwells. All measurements were performed in triplicate ($n = 3$ independent experiments), and the data are displayed as mean ± s.d. in **a–d**. Source data are provided as a Source Data file.

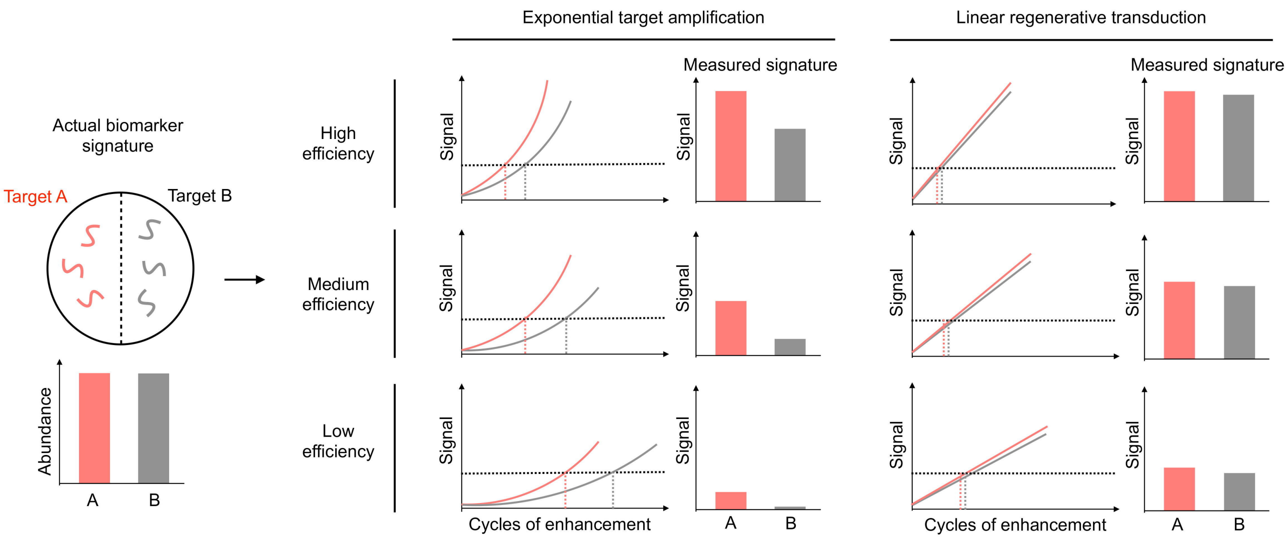


**Supplementary Figure 2. Comparison with conventional target amplification analysis.** Conventional approaches (e.g., PCR) require multiple processing steps to achieve exponential target amplification. As the amplification efficiencies are influenced by various factors (e.g., inhibitors in biological samples), small variances in the amplification efficacies are exponentially compounded, leading to large differences in the final measurements. This is especially true for PCR detection of miRNA, as short miRNA molecules need to be elongated and pre-amplified before PCR detection. On the other hand, EZ-READ utilizes linear regenerative transduction to directly release HRP@ZIF-8 nanoparticles upon target RNA hybridization. These liberated particles possess strong enzyme activity to achieve catalytic digital quantification. The approach is thus robust against efficiency fluctuations, can be applied to diverse RNA subtypes (e.g., miRNA and mRNA) and achieves direct and reflective quantification of biomarker signatures.

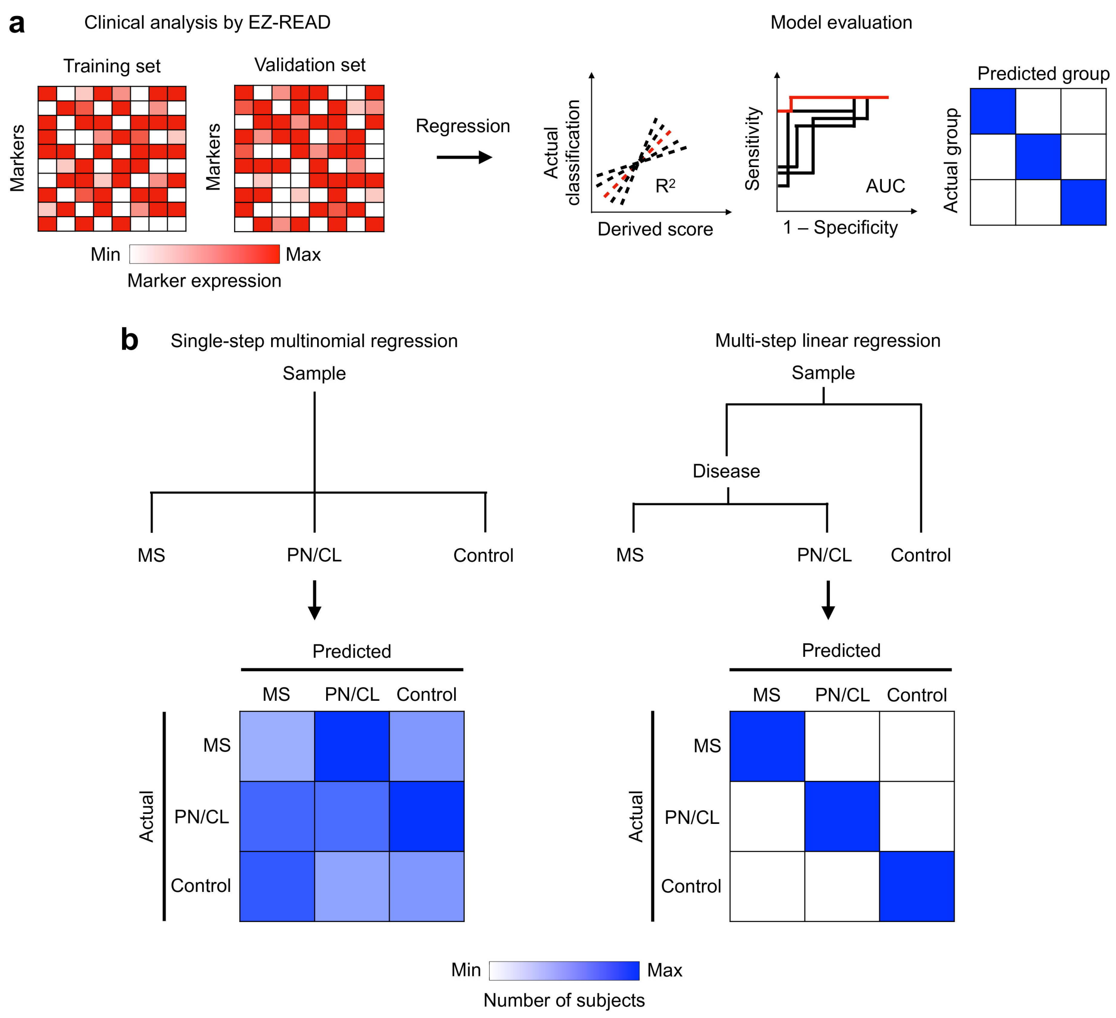


**Supplementary Figure 3. Schematics of EZ-READ clinical analysis by regression methodologies.**

**(a)** EZ-READ clinical measurements and evaluation of regression models. The EZ-READ platform was used to measure circulating RNA markers in GBM and control plasma samples. Using a training cohort of clinical samples, single-step multinomial regression and multi-step multiple linear regression scoring models were developed for the classification of disease diagnosis as well as disease subtypes. The model performance was independently validated in a separate validation patient cohort, using area under the curve (AUC) in a receiver operating characteristic (ROC) analysis. **(b)** Schematics of the single-step multinomial regression and multi-step multiple linear regression scoring models. MS, mesenchymal subtype; PN, proneural; CL, classical.

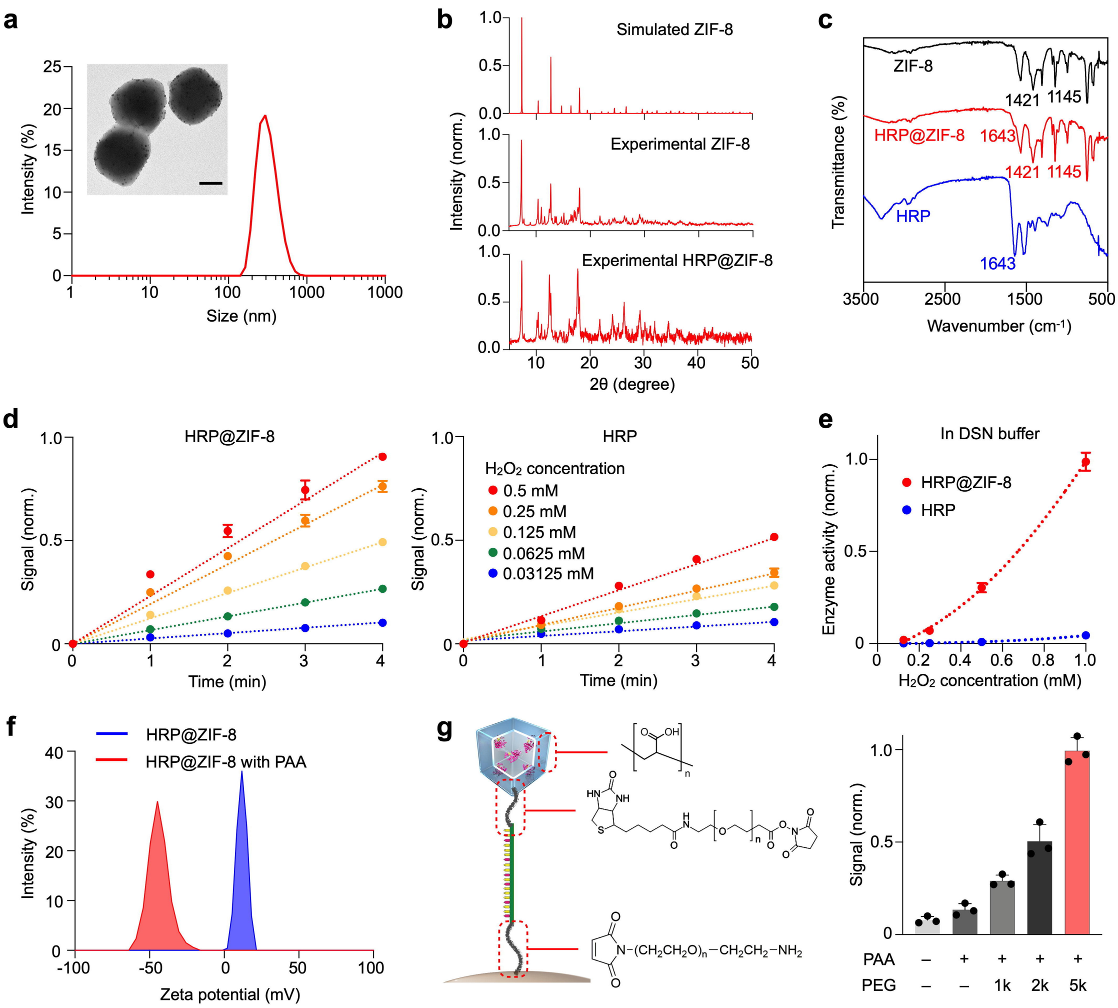


**Supplementary Figure 4. Characterization of HRP@ZIF-8 hybrid complexes.**

**(a)** Hydrodynamic diameter of the synthesized HRP@ZIF-8 nanoparticles. Dynamic light scattering analysis was used to determine the hydrodynamic diameter. Inset shows a transmission electron micrograph of the prepared HRP@ZIF-8 nanoparticles. HRP was embedded in ZIF-8 and immunolabeled with 10-nm gold nanoparticles. Scale bar: 100 nm. This experiment was repeated thrice independently with similar results. **(b)** In comparison to the simulated XRD spectrum of ZIF-8, experimental measurements of ZIF-8 and HRP@ZIF-8 showed corresponding XRD peaks, confirming the crystallinity of the prepared HRP@ZIF-8. **(c)** Fourier transform infrared (FT-IR) spectra of ZIF-8, HRP, and HRP@ZIF-8. HRP@ZIF-8 nanoparticles showed multiple characteristic peaks, thereby confirming their hybrid nature. **(d)** Enhancement of enzyme activity. HRP@ZIF-8 and equal concentration of HRP were treated with increasing concentration of hydrogen peroxide. HRP@ZIF-8 showed improved enzyme kinetics across different substrate concentrations. **(e)** Preservation of enzyme activity. When incubated in DSN buffer, HRP@ZIF-8 retained strong enzyme activity while bare HRP showed minimal activity. **(f)** Modulation of zeta potential. To reduce nonspecific binding of negatively charged DNA probes to HRP@ZIF-8, the synthesized particles were treated with polyacrylic acid (PAA) to decrease their zeta potential from 11.7 mV to –44 mV. **(g)** Tuning of PAA and PEG linkers. To enhance the transduction, PEG linkers were added to reduce steric hindrance and improve target access. When treated with target RNA, transducers prepared with both PAA coating and PEG linkers showed the highest transduction and liberation of HRP@ZIF-8 nanoparticles. All measurements were performed in triplicate (*n* = 3 independent experiments), and the data are displayed as mean ± s.d. in **d–e** and **g**. Source data are provided as a Source Data file.

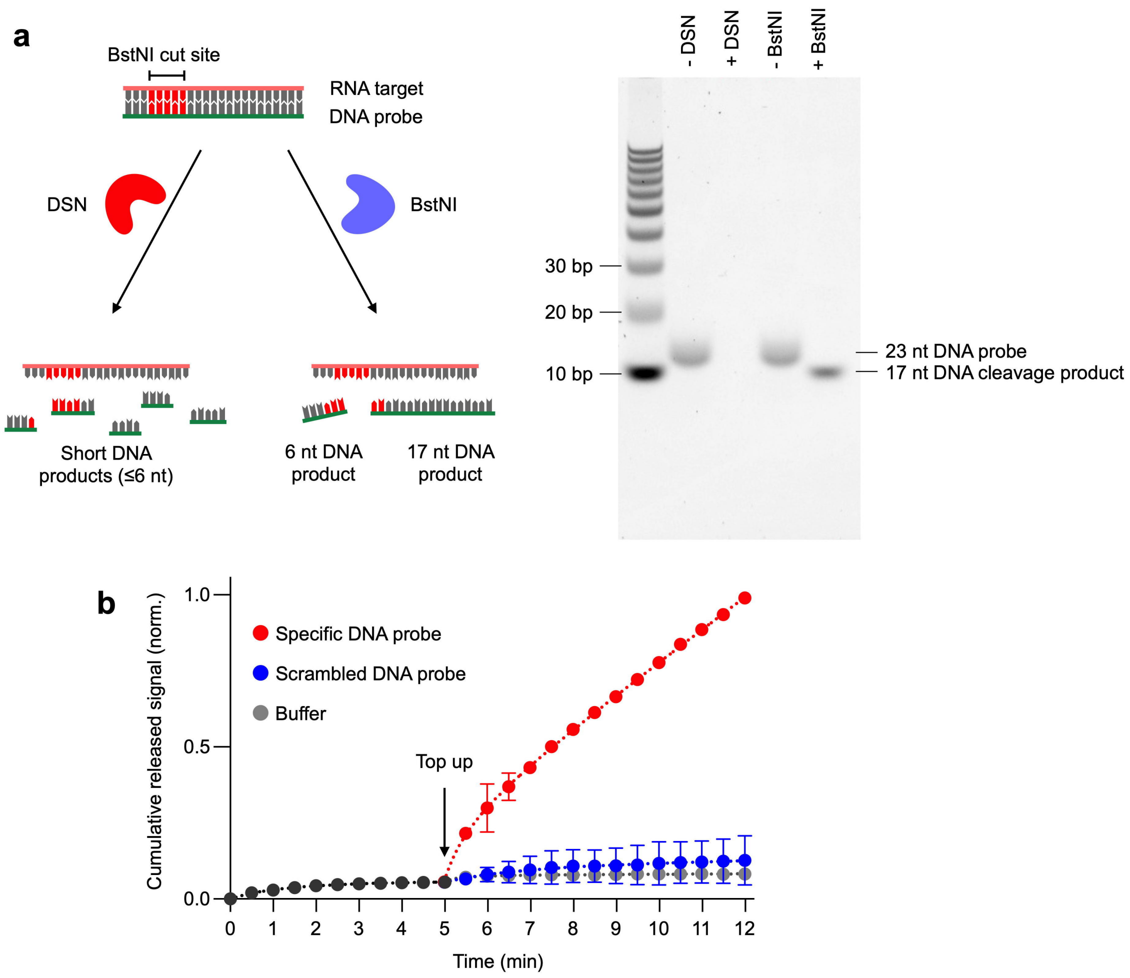


**Supplementary Figure 5. RNA target recycling.**

**(a)** Schematic illustration of enzyme mechanism differences (left). DSN recognizes the RNA-DNA heteroduplex and cleaves the DNA strand in a random, repeated manner to yield short DNA products (≤6 nt). BstNI, as an example of enzymes that mediate sequence-specific cleavage, recognizes specific cut site (CC/WGG) in the RNA-DNA heteroduplex to cleave the DNA into distinct products. Experimental validation (right). We incubated identical RNA-DNA heteroduplexes with DSN or BstNI and characterized the DNA products through polyacrylamide gel electrophoresis (10% PAGE). DSN yielded no distinct DNA product while BstNI produced a distinct band that corresponded to the specific BstNI cleavage sequence (17 nt DNA product). Note that DNA products ≤6 nt are too short for the PAGE visualization. This experiment was repeated thrice independently with similar results. **(b)** Two-step sequential reaction to validate target recycling. In the first step, RNA-DNA heteroduplexes (made from mixing RNA targets and DNA probes in a 1:1 ratio) were reacted with excess DSN. The resultant cumulative signal was measured over time and it showed quick plateau and low saturation signal. In the second step, after signal saturation, we topped up the reaction with excess specific DNA probes, scrambled DNA probes, or buffer. Only the addition of specific DNA probes resumed signal generation and attained a high signal while the scrambled control and buffer control generated minimal signal increase. The observed signal increase is thus primarily due to RNA target recycling, as the the signal output is determined by the amount of DNA probes available to form heteroduplexes with a fixed input of RNA targets. All measurements were performed in triplicate ($n = 3$ independent experiments), and the data are displayed as mean ± s.d. in **b**. Source data are provided as a Source Data file.

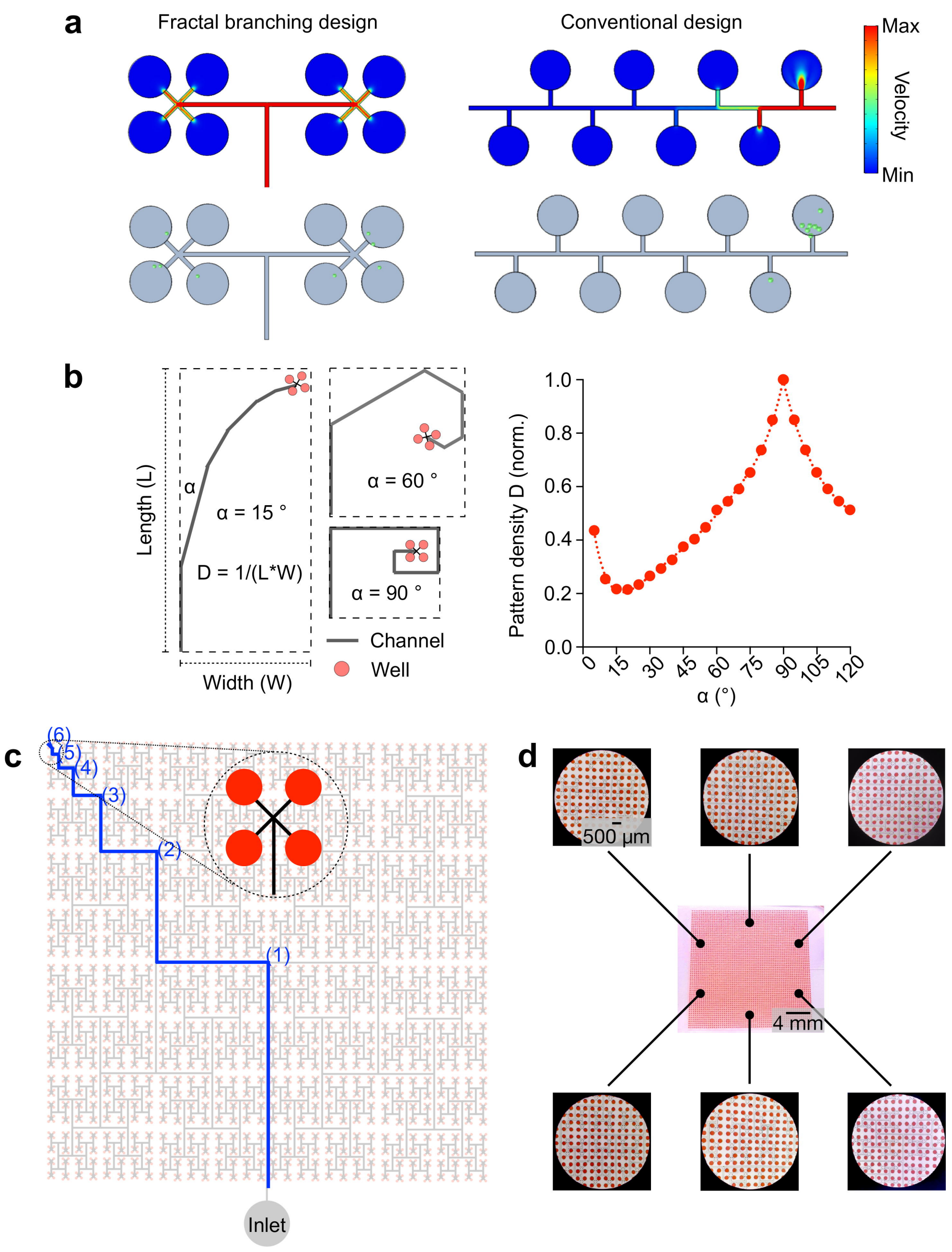


**Supplementary Figure 6. Fractal branching microfluidics design and characterization.** **(a)** Two-dimensional fluid simulation diagram and particle tracking analysis of the fractal branching design (left) and conventional sequential layout (right). The fractal branching design showed more uniform flow velocity and even particle compartmentalization. **(b)** Optimization of branch angle. Different branch angles (α = 15 °, 60 ° and 90 °) resulted in different microwell density. Theoretical calculation showed that α = 90 ° achieved a high pattern density. **(c)** Schematic of the optimized microfluidics. The chip features six layers of fractal branching, where the main fluidic channel is repeatedly divided and subdivided into thousands of branches. Each branch is terminally connected to four microwells. This design thus achieves equal fluidic length and resistance to individual microwells, facilitates even microwell loading, and enables scalable expansion. **(d)** Large-scale device image. Magnified views over different spatial regions demonstrate uniform reagent loading and even distribution in individual microwells. This experiment was repeated thrice independently with similar results. Source data are provided as a Source Data file.

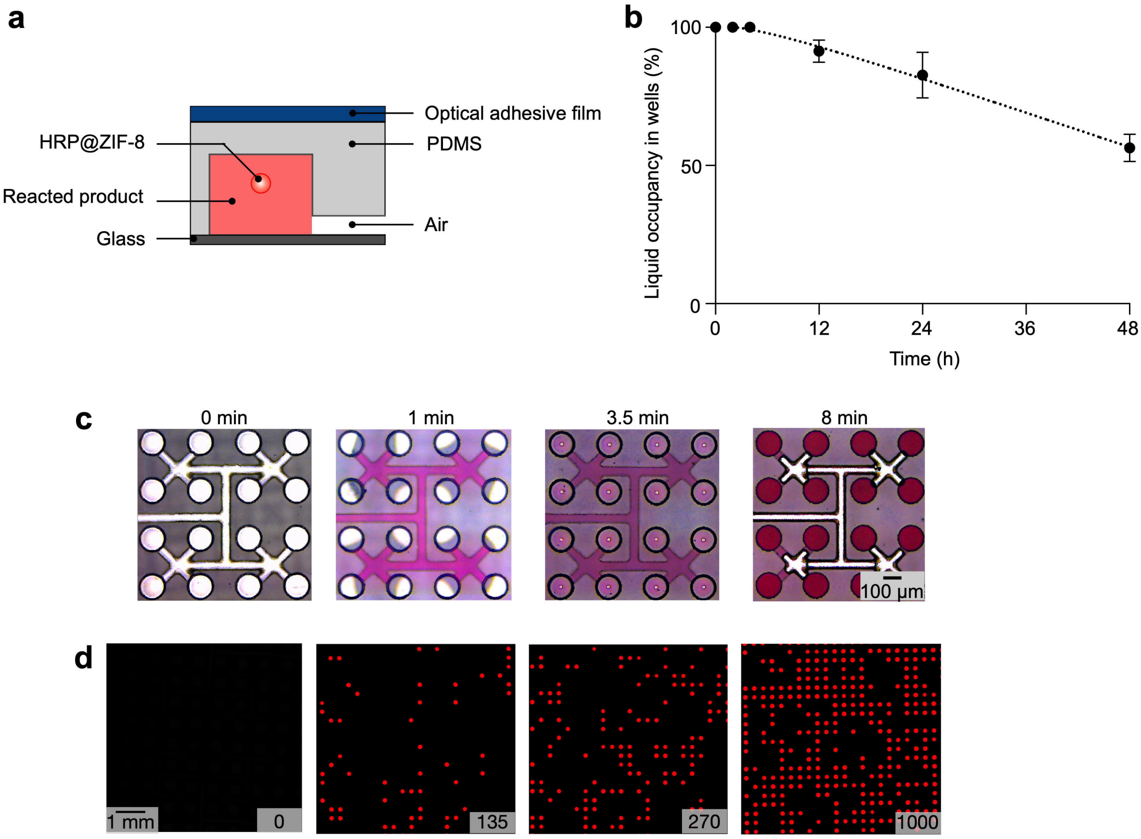


**Supplementary Figure 7. Liquid occupancy characterization.**
**(a)** Schematic of the filmed device. The PDMS chamber was covered with an impermeable, optical adhesive film to reduce liquid evaporation. **(b)** Experimental characterization. We monitored the liquid occupancy in the microwells. High liquid occupancy could be maintained for at least several hours, which is significantly longer than the assay duration (~30 min). **(c)** Sequential solution loading. The microfluidic platform supported simultaneous microwell filling and effective air sealing of individual reaction chambers. This experiment was repeated thrice independently with similar results. **(d)** Reflective nanoparticle quantification. Different numbers of HRP@ZIF-8 nanoparticles were introduced into the microfluidic chip. After loading of chemifluorescence substrate and air for effective sealing, end-point fluorescence images were recorded. This experiment was repeated thrice independently with similar results. All measurements were performed in triplicate ($n = 3$ independent experiments), and the data are displayed as mean ± s.d. in **b**. Source data are provided as a Source Data file.

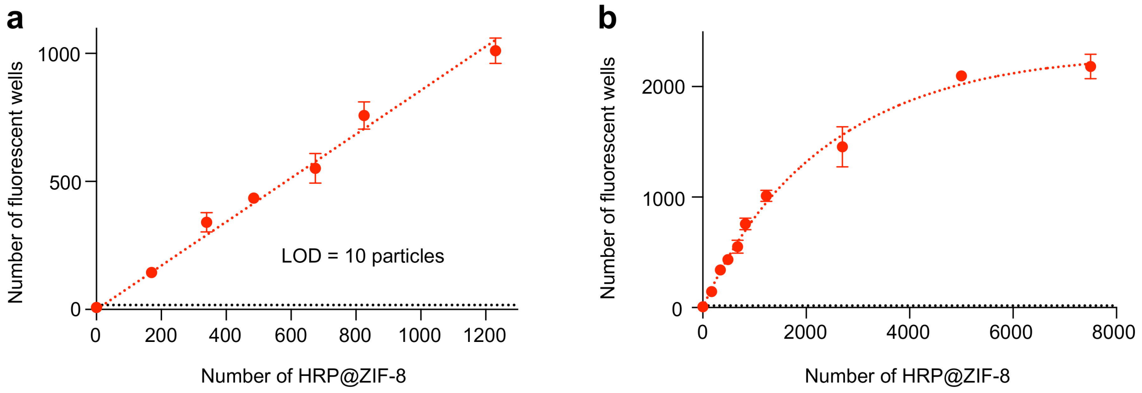


**Supplementary Figure 8. Quantification of HRP@ZIF-8 nanoparticles.**
**(a)** The limit of detection (LOD, dotted line) was determined ~10 particles, as defined by 3 × s.d. of a matched control (no particle control). **(b)** The maximum detection limit was estimated ~4000 particles based on the dynamic distinguishable signal range. All measurements were performed in triplicate ($n = 3$ independent experiments), and the data are displayed as mean ± s.d. Source data are provided as a Source Data file.

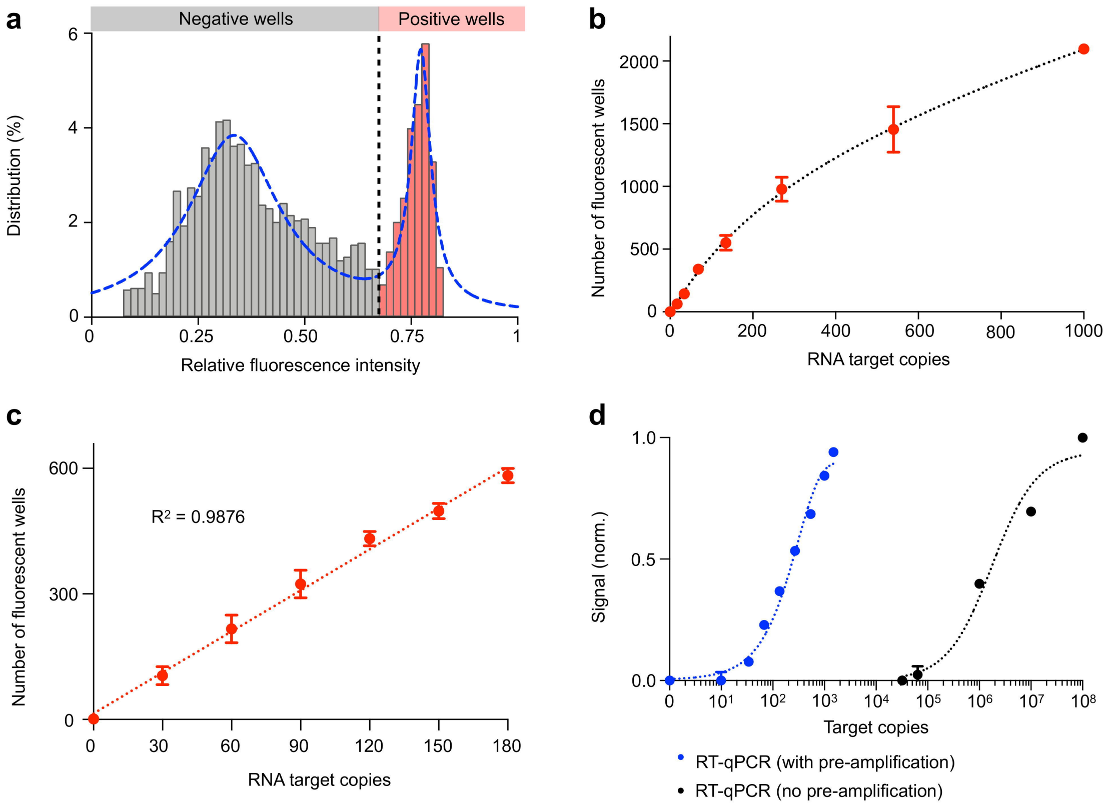


**Supplementary Figure 9. EZ-READ performance evaluation.**
**(a)** Threshold determination for positive wells. Fluorescence distribution of microwells showed two distinguishable peaks. Positive wells were determined by setting a threshold to eliminate the background caused by substrate autofluorescence. **(b)** Correlation of positive microwells and target RNA molecules. The number of fluorescent wells showed a good correlation with target abundance. **(c)** Calibration curve for absolute quantification of RNA levels. Human plasma was spiked with varying amounts of *Arabidopsis thaliana ath-miR159a*, a target absent in human samples. The spiked plasma was incubated with transducers against *ath-miR159a*, and the number of fluorescent wells was determined to establish a calibration curve for target quantitation. **(d)** Sensitivity of RT-qPCR for miRNA detection. The limit of detection (LOD) was determined by titrating a known amount of *miR-222-3p* target. The miRNA sample was reverse transcribed and subjected to either qPCR directly (without pre-amplification) or pre-amplification processing and subsequent PCR amplification (with pre-amplification before qPCR analysis). The LOD is defined as 3 × s.d. of a no-target control. $n = 3$ independent experiments in **b–d**. All measurements were performed in triplicate ($n = 3$ independent experiments), and the data are displayed as mean ± s.d. in **b–d**. Source data are provided as a Source Data file.

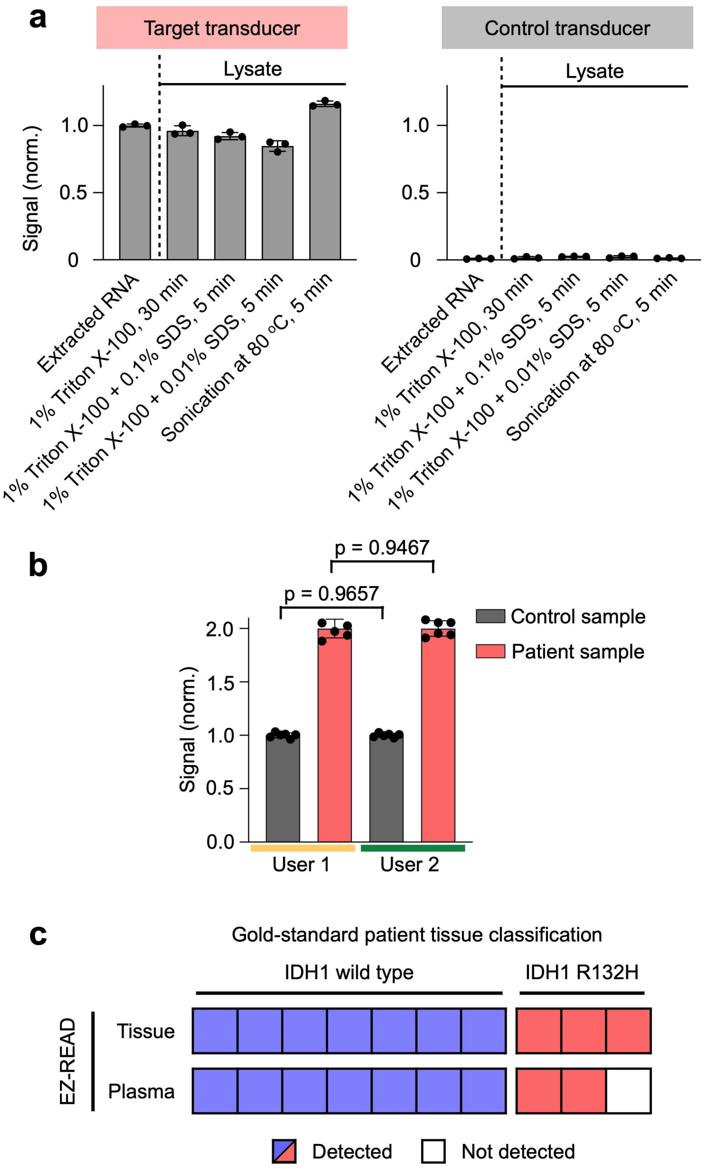


**Supplementary Figure 10. Analytical evaluation of EZ-READ.**
**(a)** EZ-READ quantification of RNA target in lysed biological samples. GLI36vIII cell lysates were prepared through chemical lysis and sonication, respectively, without RNA purification. Using the designed target transducer, the EZ-READ assay was able to detect endogenous *miR-222-3p* in these samples prepared by chemical lysis and sonication. It generated comparable signals to that measured in the gold standard extracted RNA. The control transducer, against *Arabidopsis thaliana ath-miR159a*, generated negligible signals. Measurements were performed in triplicate ($n$ = 3 independent experiments), and the data are displayed as mean ± s.d. **(b)** EZ-READ analytical validation. The same samples were measured by different users on different EZ-READ chips. The technology showed robust performance with small coefficients of variation (within user = 2.04% and between users = 3.83%). $n$ = 6 technical replicates. Data are displayed as mean ± s.d. Two-sided Student's t test was performed with adjustment for multiple comparisons using Bonferroni correction. **(c)** EZ-READ analysis of IDH1 mutation status. Tumor tissue and paired plasma samples ($n$ = 10 patients, 7 with *IDH1* wild type and 3 with *IDH1 R132H* mutation) were analyzed through the EZ-READ platform. Gold standard patient classification was performed through tumor tissue immunohistochemistry and sequencing. EZ-READ analysis of tumor tissue and plasma achieved an overall accuracy of 100% and 90%, respectively. Measurements were performed in triplicate ($n$ = 3 independent experiments). Source data are provided as a Source Data file.

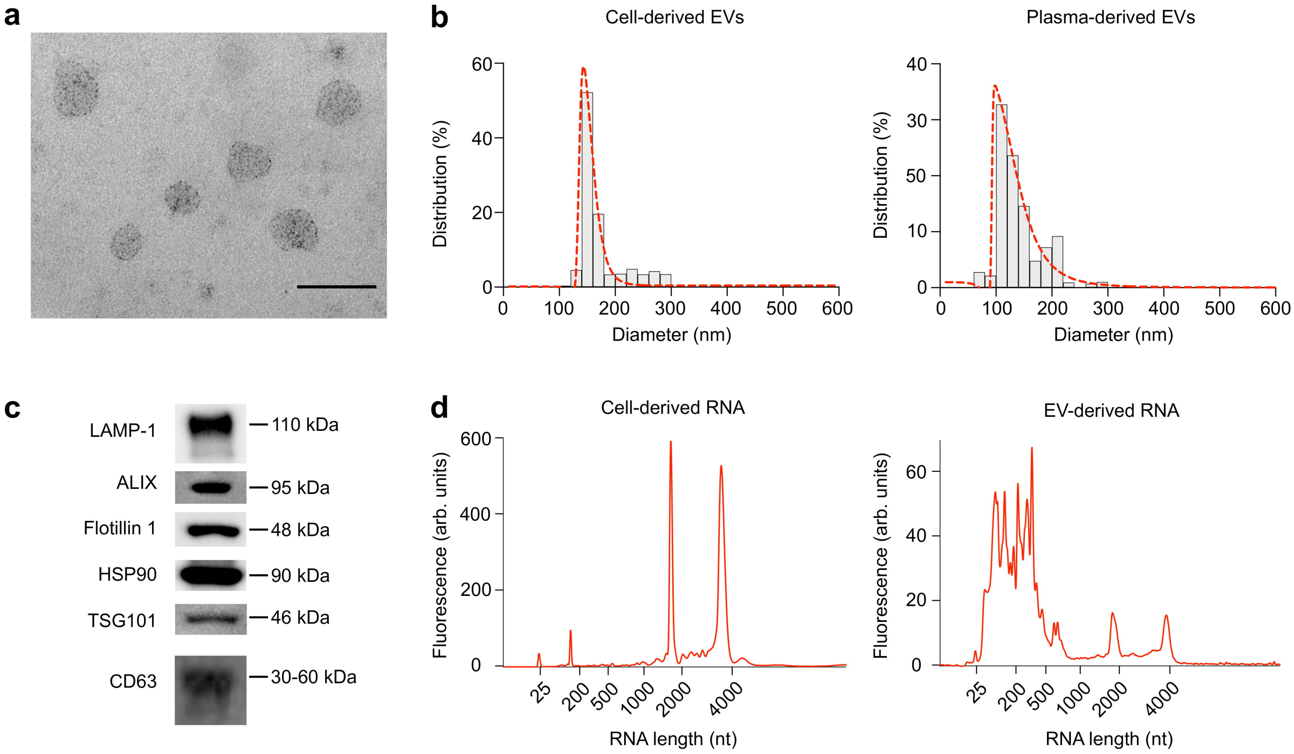


**Supplementary Figure 11. Molecular characterization of EVs.**
**(a)** Transmission electron micrograph of EVs from GLI36vIII cells. Scale bar: 100 nm. **(b)** Unimodal size distribution of EVs derived from cell line (left) and plasma (right), as determined by nanoparticle tracking analysis, showing similar mean diameter of ~130 nm. A total of 6.69 x $10^8$ and 3.22 x $10^9$ vesicles were measured for histogram analysis of cell-derived EVs and plasma-derived EVs, respectively. **(c)** Western blotting analysis of GLI36vIII EV lysate. EVs were lysed and immunoblotted for markers enriched in EVs, especially for exosomal biomarkers. Molecular weight markers are provided with the full blots as a Source Data file. **(d)** Bioanalyzer electropherograms showed good structural integrity and quality profile of RNA extracted from GLI36vIII cells (left) and EVs (right). RNA obtained from EVs showed higher miRNA:mRNA ratio than cellular RNA. All experiments were repeated thrice independently with similar results. Source data are provided as a Source Data file.

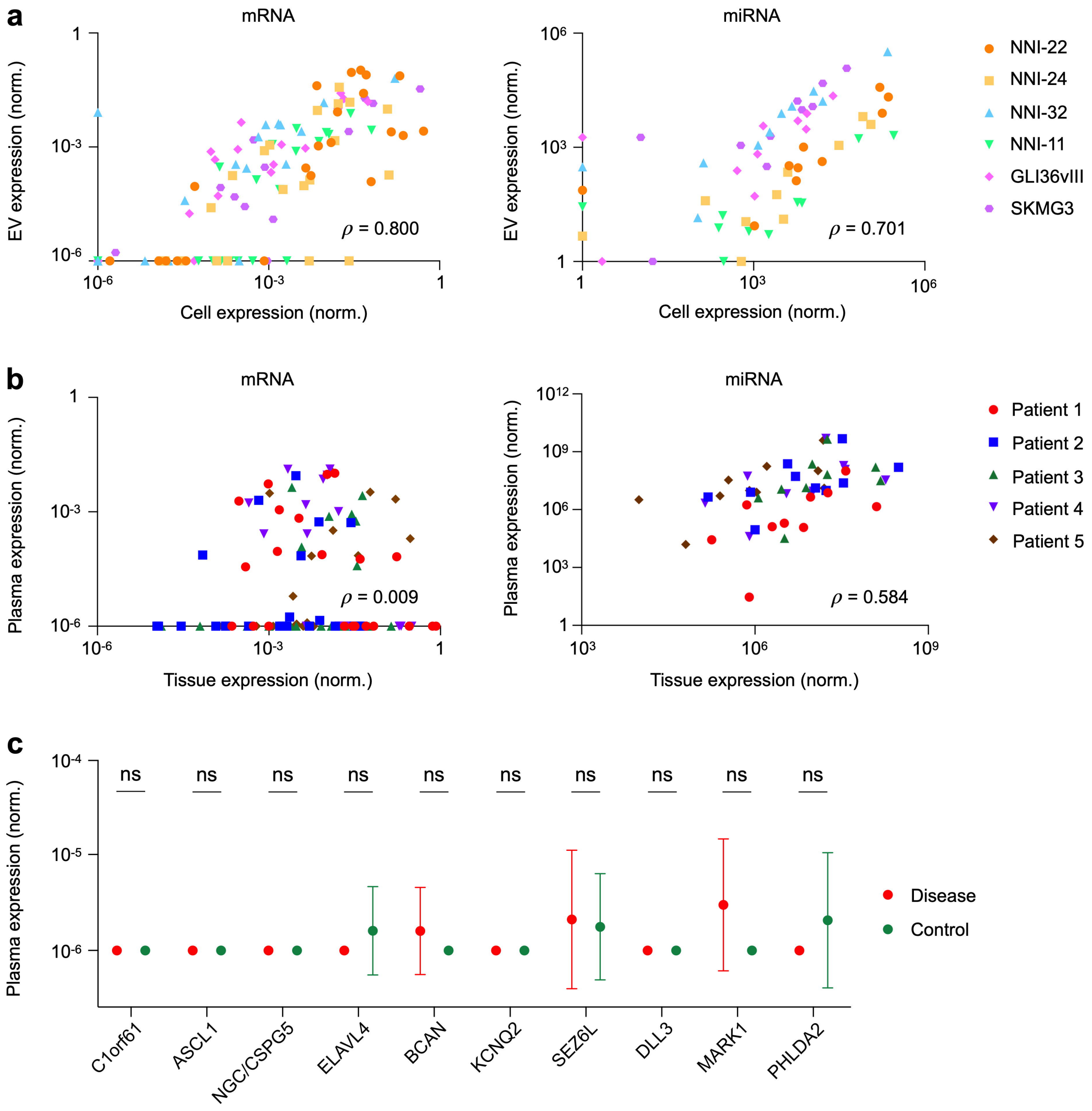


**Supplementary Figure 12. Selection of mRNA and miRNA markers.**

**(a)** Quantification of mRNA and miRNA in cell lines and derived EVs. Cell lines and derived EVs showed a good expression correlation for the measured mRNA and miRNA markers (two-sided Spearman's ρ = 0.800 and 0.701 respectively). **(b)** Quantification of mRNA and miRNA in tumor tissues and matched plasma-derived EVs. There was a poor correlation between tumor tissues and matched plasma for mRNA and miRNA markers (two-sided Spearman's ρ = 0.009 and 0.584 respectively). **(c)** Comparison between eliminated markers in disease and control plasma samples. No significant differences were observed for these mRNA targets in disease plasma samples ($n$ = 5) vs. control plasma samples ($n$ = 5) for markers. All measurements were performed in triplicate ($n$ = 3 independent experiments). mRNA levels were normalized to intrinsic GAPDH expression and miRNA levels were normalized to total RNA amount. Data are displayed as mean ± s.d. in **c**. ns = not significant, two-sided Student's t test.

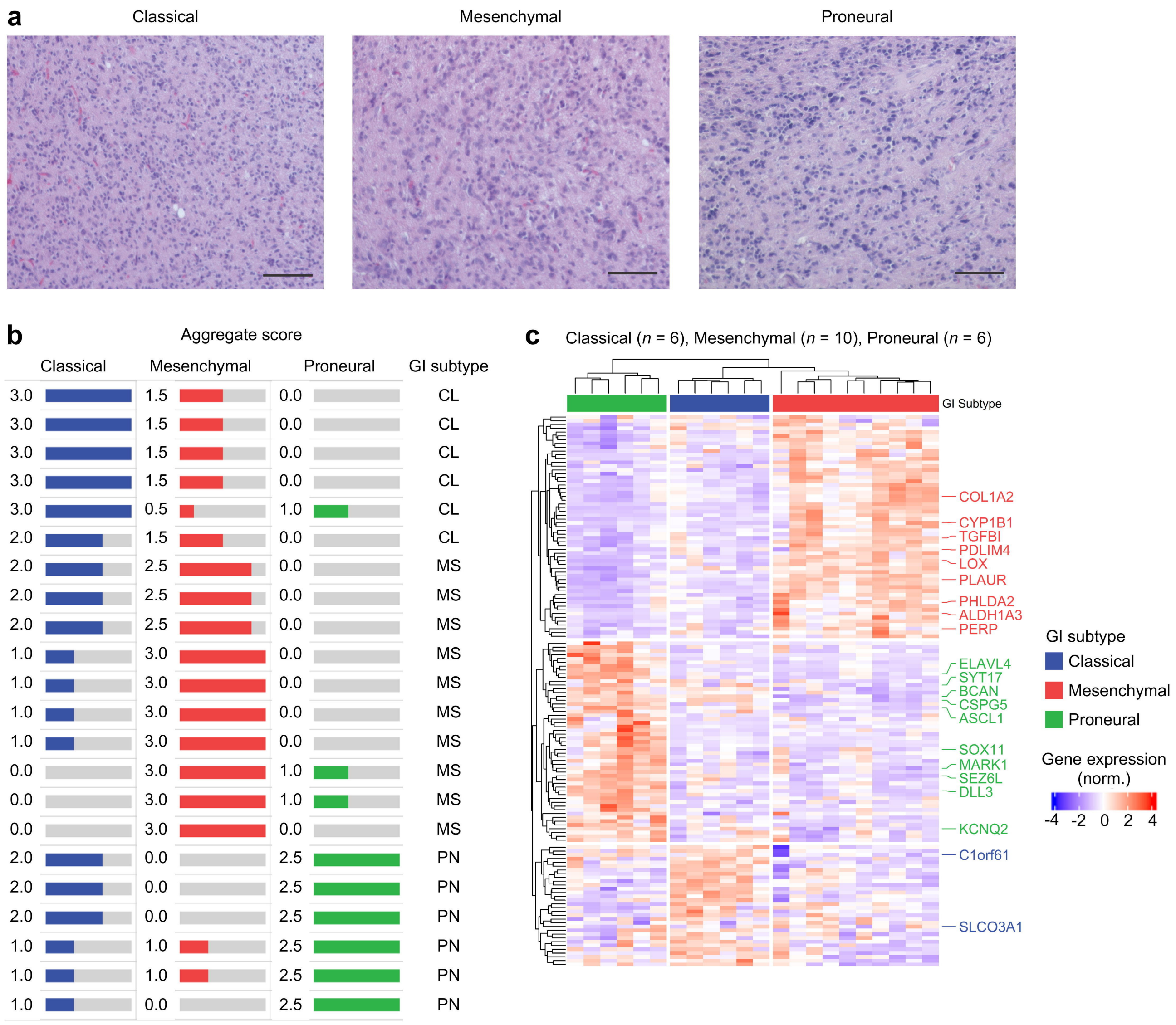


**Supplementary Figure 13. Marker validation in clinical tumor tissues.**

**(a)** Representative images of classical, mesenchymal and proneural transcriptomic subtypes of GBM tumors demonstrating similar morphology. Scale bar: 100 µm. This experiment was repeated thrice independently with similar results. **(b)** Weighted aggregate scores for glioma-intrinsic (GI) classification by RNA sequencing analysis. Single-sample Gene Set Enrichment Analysis (ssGSEA) was carried out using GI signatures for GBM tumor samples to assign the tumor subtypes. **(c)** Hierarchical clustering of 22 GBM tumors with expression levels of cross-validation threshold-filtered genes and EZ-READ genes showed three distinct clusters corresponding to the GI subtypes.

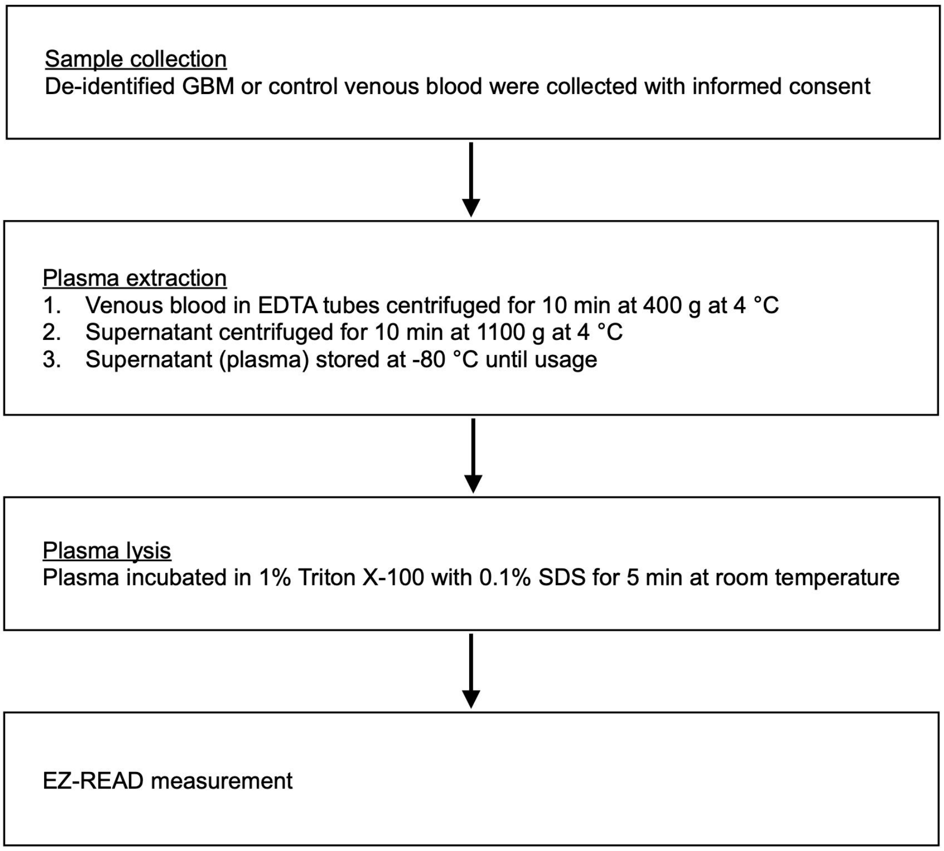


**Supplementary Figure 14. Clinical sample workflow.**
Clinical samples were first collected and processed for plasma extraction. Plasma samples were lysed prior to EZ-READ measurement.

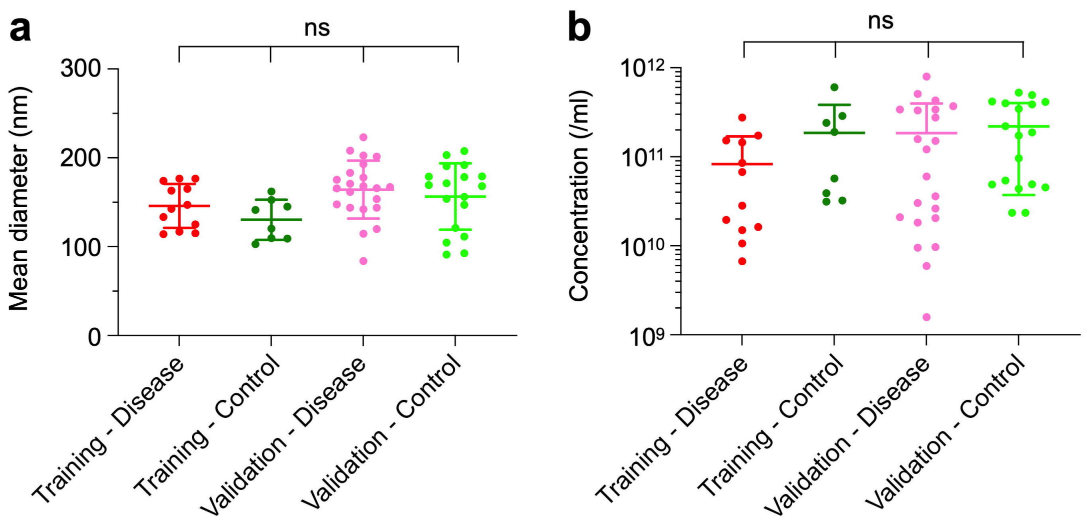


**Supplementary Figure 15. Characterization of clinical plasma samples.**

Neither **(a)** the mean diameters nor **(b)** the concentrations of plasma EVs showed any statistical significance among the different groups of clinical samples (*n* = 60 subjects; training cohort comprised 12 disease and 8 control, validation cohort comprised 22 disease and 18 control). Clinical information is found in **Supplementary Table 3**. All measurements were performed in triplicate (*n* = 3 independent experiments), and the data are displayed as mean ± s.d. ns = not significant, two-sided Student's t test.

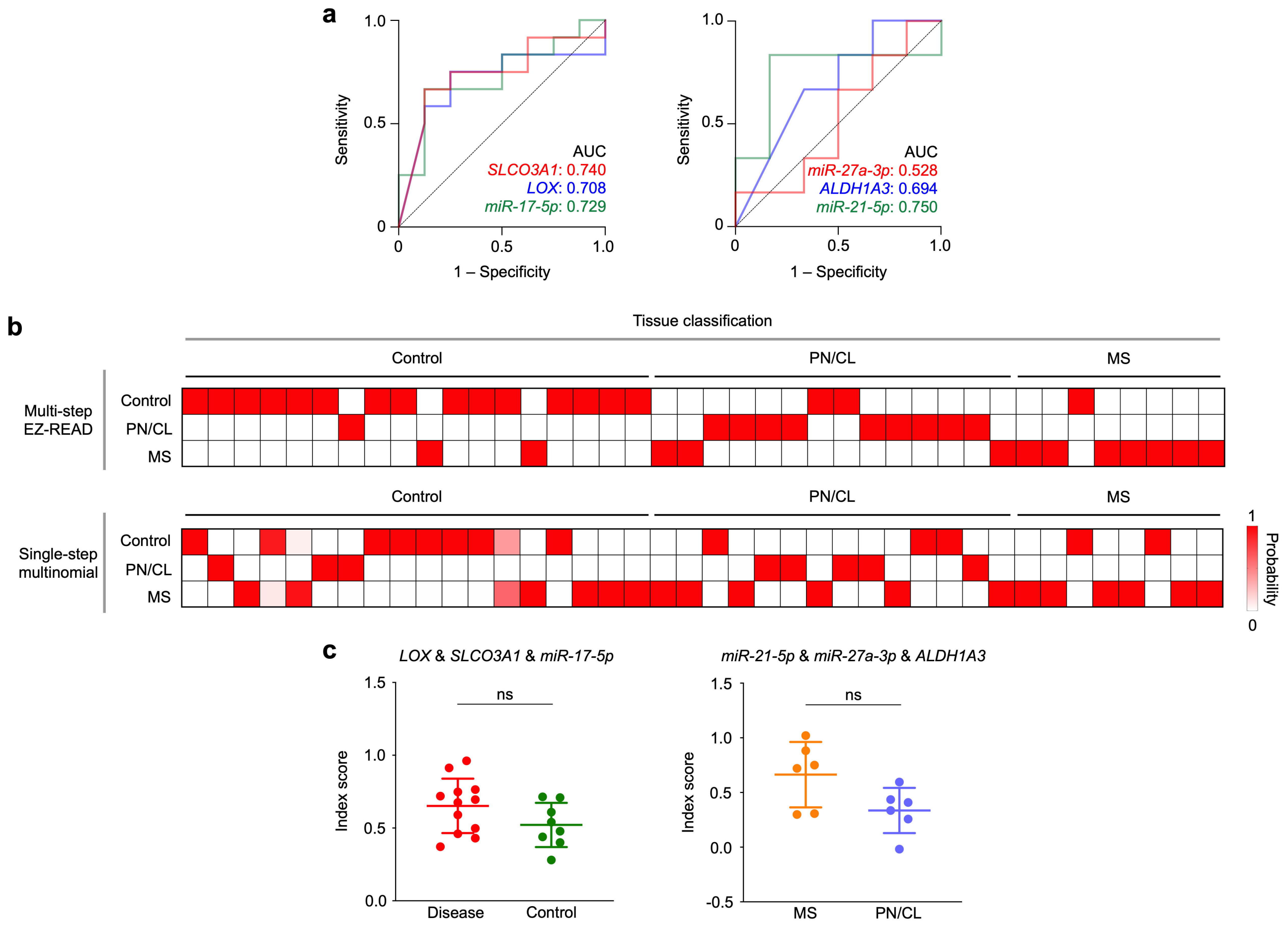


**Supplementary Figure 16. Multi-step decision model.**

**(a)** Performance of multi-step linear regression with individual markers. Receiver operating characteristic (ROC) curves with individual markers for the Disease (left) and Mesenchymal (right) indices with the training set. Note that the area under curve (AUC) by the individual markers are lower than that by the composite marker panels. **(b)** Comparison against single-step multinomial regression. Using the six derived markers (*miR-17-5p*, *miR-21-5p*, *miR-27a-3p*, *LOX*, *ALDH1A3* and *SLCO3A1*), the multi-step EZ-READ model was able to accurately classify the validation clinical samples into control, proneural/classical and mesenchymal samples (78% accuracy). The single-step multinomial regression model was unable to accurately classify the validation samples into control, proneural/classical and mesenchymal samples (48% accuracy). **(c)** RT-qPCR quantification and model classification for Disease (left) and Mesenchymal (right). Multiple linear regressions with the PCR-measured markers were unable to classify the disease and control samples nor distinguish the mesenchymal samples from others. $n$ = 20 subjects for disease classification and $n$ = 12 subjects for mesenchymal classification in **c**. All measurements were performed in triplicate ($n$ = 3 independent experiments), and the data are displayed as mean ± s.d. in **c**. ns = not significant, two-sided Student's t test. For disease classification, P = 0.1170. For mesenchymal classification, P = 0.0518. Source data are provided as a Source Data file.

**Supplementary Table 1. Sequences for validation of DSN cleavage mechanism.**

| Oligonucleotide | Sequence (5’ to 3’) |
|---|---|
| DNA probe | AGGTAGACAAGTGCTCCAGGTGC |
| RNA target | GCACCUGGAGCACUUGUCUACCU |

**Supplementary Table 2. Probe designs and sequences for EZ-READ evaluation.**
Red nucleotides indicate the locations of mismatches.

| Oligonucleotide | Sequence (5' to 3') |
|---|---|
| *miRNA-222-3p* EZ-READ probe | /5ThioMC6-D/ACCCAGTAGCCAGATGTAGCT/3AmMO/ |
| *miRNA-222-3p* RNA target | AGCUACAUCUGGCUACUGGGU |
| *miRNA-222-3p* with 2 mismatches | AGCUACAUCAGGCAACUGGGU |
| *miRNA-222-3p* with 4 mismatches | AGCUAGAUCAGGCAACUCGGU |
| *miRNA-222-3p* with 6 mismatches | AGGUAGAUAUGCCUGCUCGGU |
| *LOX* EZ-READ probe | /5ThioMC6-D/AGTTTCACGGCTGCCTTATGTA/3AmMO/ |
| *LOX* RNA target | CUGGUAUACAUAAGGCAGCCGUGAAACUGGAAAGU |
| *SLCO3A1* EZ-READ probe | /5ThioMC6-D/ACATTGTCGTAGAGGACGCA/3AmMO/ |
| *SLCO3A1* RNA target | AAGGCGCCUGCGUCCUCUACGACAAUGUGGUCUAC |
| *COL1A2* EZ-READ probe | /5ThioMC6-D/CCTTTTCCATCATACTGAGCAGC/3AmMO/ |
| *COL1A2* RNA target | AACUUUGCUGCUCAGUAUGAUGGAAAAGGAGUUGG |
| *miRNA-17-5p* EZ-READ probe | /5ThioMC6-D/CTACCTGCACTGTAAGCACTTTG/3AmMO/ |
| *miRNA-17-5p* target | CAAAGUGCUUACAGUGCAGGUAG |
| *miRNA-21-5p* EZ-READ probe | /5ThioMC6-D/TCAACATCAGTCTGATAAGCTA/3AmMO/ |
| *miRNA-21-5p* target | UAGCUUAUCAGACUGAUGUUGA |
| *miRNA-27a-3p* EZ-READ probe | /5ThioMC6-D/CGCGGAACTTAGCCACTGTGAA/3AmMO/ |
| *miRNA-27a-3p* target | UUCACAGUGGCUAAGUUCCGC |
| *IDH1* wild type EZ-READ probe | /5ThioMC6-D/ATAAGCATGACGACCTATGAT/3AmMO/ |
| *IDH1 R132H* EZ-READ probe | /5ThioMC6-D/ATAAGCATGATGACCTATGAT/3AmMO/ |
| *ath-miR159a* EZ-READ control probe | /5ThioMC6-D/TAGAGCTCCCTTCAATCCAAA/3AmMO/ |
| *ath-miR159a* control target | UUGGAUUGAAGGGAGCUCUA |

**Supplementary Table 3. Clinical characteristics of GBM patients and control subjects.**

| Characteristics | Training | | Validation | | Total (*n* = 60) | |
|---|---|---|---|---|---|---|
| | GBM | Control | GBM | Control | GBM | Control |
| Individuals | 12 | 8 | 22 | 18 | 34 | 26 |
| Median age, years (IQR) | 57 (38.5-62.5) | 55.5 (39.5-61.5) | 54.5 (45-69) | 59 (43-67) | 54.5 (44-67) | 59 (43-63) |
| Sex | | | | | | |
| - Male | 6 | 4 | 17 | 11 | 23 | 15 |
| - Female | 6 | 4 | 5 | 7 | 11 | 11 |
| Subtype (%) | | | | | | |
| - Mesenchymal | 6 (50%) | - | 8 (36%) | - | 14 (41%) | - |
| - Proneural | 3 (25%) | - | 7 (32%) | - | 10 (29%) | - |
| - Classical | 3 (25%) | - | 7 (32%) | - | 10 (29%) | - |
| Co-morbidity (%) | | | | | | |
| - Diabetes | 1 (8%) | - | 4 (18%) | - | 5 (15%) | - |
| - Hypertension | 3 (25%) | - | 8 (36%) | - | 11 (32%) | - |
| - Hyperlipidemia | 2 (17%) | - | 5 (23%) | - | 7 (21%) | - |
| Treatment regimen (%) | | | | | | |
| - Radiotherapy | 7 (58%) | - | 12 (55%) | - | 19 (56%) | - |
| - Chemotherapy | 6 (50%) | - | 11 (50%) | - | 17 (50%) | - |

**Supplementary Table 4. Comparison with FRET-based systems for miRNA detection.**

| | EZ-READ | FRET without amplification | FRET with amplification | |
|---|---|---|---|---|
| | | Hybridization (Molecular beacon)[1-3] | Isothermal amplification (RT-LAMP)[4,5] | RT-qPCR (TaqMan/ SYBR Green)[6-8] |
| Detection mechanism | Direct detection of RNA with linear signal amplification based on regenerative transduction of HRP@ZIF-8 nanoparticles | Direct detection of RNA without amplification based on fluorescence restoration of internally quenched fluorophore | Isothermal exponential amplification of cDNA with detection of fluorescence signal via hydrolysis probes or intercalating dyes | Thermal cycling-based exponential amplification of cDNA with detection of fluorescence signal via hydrolysis probes or intercalating dyes |
| Sample processing requirement | Low | Moderate *(RNA extraction)* | High *(RNA extraction, reverse transcription)* | High *(RNA extraction, reverse transcription)* |
| Amplification condition | Isothermal | Isothermal | Isothermal | Thermal cycling |
| Compatibility for short RNA | High *(direct detection)* | High *(direct detection)* | Low *(extensive processing to incorporate additional sequence)* | Low *(extensive processing to incorporate additional sequence)* |
| Sequence design stringency | Low *(single sequence)* | Low *(single sequence)* | High *(sets of four or six primer sequences)* | Moderate *(sets of two or three primer and probe sequences)* |
| Detection limit (copies) | ~9 | 3000 — $3 \times 10^8$ | $6 \times 10^5$ — $8.4 \times 10^8$ | ~7 — $1.2 \times 10^6$ |
| Time taken | ~30 min | ~1 h | 1 — 2 h | 2 — 4 h |
| Equipment requirement | Moderate | Moderate | Moderate | High |

## Supplementary References


1. Caputo, T.M., Battista, E., Netti, P.A. and Causa, F. *ACS Appl. Mater. Interfaces*, *11*(19), 17147-17156 (2019).
2. Hwang, J.Y. et al. *ACS Sens.*, *3*(12), 2651-2659 (2018).
3. Hu, J. et al. *Biomaterials*, *183*, 20-29 (2018).
4. Li, C., Li, Z., Jia, H. and Yan, J. *Chem. Commun.*, *47*(9), 2595-2597 (2011).
5. Williams, M.R., Stedtfeld, R.D., Stedtfeld, T.M., Tiedje, J.M. and Hashsham, S.A. *Biomed. Microdevices*, *19*, 1-8 (2017).
6. Chen, C. et al. *Nucleic Acids Res.*, *33*(20), e179-e179 (2005).
7. Jung, S. et al. *Biosens. Bioelectron.*, *163*, 112301 (2020).
8. Ge, Q. et al. *Anal. Methods*, *6*(22), 9101-9107 (2014).
