# Supplementary material for: Multiplexed RNA profiling by regenerative catalysis enables blood-based subtyping of brain tumors
Source: Nat Commun. 2023 Jul 17;14:4278. doi: 10.1038/s41467-023-39844-0 (PMC10352249; doi:10.1038/s41467-023-39844-0)
Supplement: Supplementary file 3 — Description of Additional Supplementary File [file 41467_2023_39844_MOESM3_ESM.pdf]

Description of Additional Supplementary File

Movie Legends:

Supplementary Movie 1. Chip operation.

Supplementary Movie 2. Fluorescence signal generation.
