# Supplementary material for: Multiplexed RNA profiling by regenerative catalysis enables blood-based subtyping of brain tumors
Source: Nat Commun. 2023 Jul 17;14:4278. doi: 10.1038/s41467-023-39844-0 (PMC10352249; doi:10.1038/s41467-023-39844-0)
Supplement: Supplementary file 6 — Reporting Summary [file 41467_2023_39844_MOESM6_ESM.pdf]

nature portfolio

Corresponding author(s): Huilin Shao

Last updated by author(s): Jun 12, 2023

Reporting Summary

Nature Portfolio wishes to improve the reproducibility of the work that we publish. This form provides structure for consistency and transparency in reporting. For further information on Nature Portfolio policies, see our Editorial Policies and the Editorial Policy Checklist.

Statistics

For all statistical analyses, confirm that the following items are present in the figure legend, table legend, main text, or Methods section.

n/a	Confirmed	
☐	☒	The exact sample size (*n*) for each experimental group/condition, given as a discrete number and unit of measurement
☐	☒	A statement on whether measurements were taken from distinct samples or whether the same sample was measured repeatedly
☐	☒	The statistical test(s) used AND whether they are one- or two-sided<br>*Only common tests should be described solely by name; describe more complex techniques in the Methods section.*
☐	☒	A description of all covariates tested
☐	☒	A description of any assumptions or corrections, such as tests of normality and adjustment for multiple comparisons
☐	☒	A full description of the statistical parameters including central tendency (e.g. means) or other basic estimates (e.g. regression coefficient) AND variation (e.g. standard deviation) or associated estimates of uncertainty (e.g. confidence intervals)
☐	☒	For null hypothesis testing, the test statistic (e.g. *F*, *t*, *r*) with confidence intervals, effect sizes, degrees of freedom and *P* value noted<br>*Give P values as exact values whenever suitable.*
☒	☐	For Bayesian analysis, information on the choice of priors and Markov chain Monte Carlo settings
☒	☐	For hierarchical and complex designs, identification of the appropriate level for tests and full reporting of outcomes
☐	☒	Estimates of effect sizes (e.g. Cohen's *d*, Pearson's *r*), indicating how they were calculated

Our web collection on statistics for biologists contains articles on many of the points above.

Software and code

Policy information about availability of computer code

Data collection	LAS X (v.3.6.123246), SparkControl v2.1, NTA v3.3, ImageJ (v.1.53k)
Data analysis	R-package v.4.0.3, STAR v2.7.9a, RSEM v1.3.3, pamr package v1.56.1, GraphPad Prism v.9.0.0

For manuscripts utilizing custom algorithms or software that are central to the research but not yet described in published literature, software must be made available to editors and reviewers. We strongly encourage code deposition in a community repository (e.g. GitHub). See the Nature Portfolio guidelines for submitting code & software for further information.

March 2021

Data

Policy information about availability of data

All manuscripts must include a data availability statement. This statement should provide the following information, where applicable:

- Accession codes, unique identifiers, or web links for publicly available datasets
- A description of any restrictions on data availability
- For clinical datasets or third party data, please ensure that the statement adheres to our policy

The main data supporting the results in this study are available within the paper and its Supplementary Information. Source data are provided with this paper. Raw sequencing data that support the findings of this study are available upon reasonable request due to patient privacy protection. Request for data access should be directed to Beng Ti Ang (ang.beng.ti@singhealth.com.sg). Review of the request will be completed within two months. Upon request approval by the National Neuroscience Institute Tissue Bank, data will be accessible for research use only. The processed RNA sequencing data can be downloaded from Synapse under the project ID: syn51691297.

Human research participants

Policy information about studies involving human research participants and Sex and Gender in Research.

Reporting on sex and gender	Subjects are sex-matched. Clinical information of subjects are provided in Supplementary Table 3.
Population characteristics	Clinical information of subjects are provided in Supplementary Table 3. Subjects were age and sex-matched, with a median age of 54.5 for GBM patients and 59 for control subjects, respectively. GBM patients clinically characterized, through pathology and RNA sequencing of the primary tumor tissue for disease subtyping.
Recruitment	No selection bias. Subjects were recruited based on clinical diagnosis as determined by independent gold-standard pathology and RNA sequencing of the primary tumor tissue. All subjects were recruited according to IRB-approved protocols after obtaining informed consent. A definitive clinical diagnosis of GBM was the only selection criterion.
Ethics oversight	This study was approved by the National University of Singapore Institutional Review Board (NUS-IRB no. 2021-152). De-identified clinical specimens were obtained with informed consent from the National Neuroscience Institute Tissue Bank (application no. SBRSA2019/002) in accordance with the SingHealth Centralized Institutional Review Board.

Note that full information on the approval of the study protocol must also be provided in the manuscript.

Field-specific reporting

Please select the one below that is the best fit for your research. If you are not sure, read the appropriate sections before making your selection.

[x] Life sciences [ ] Behavioural & social sciences [ ] Ecological, evolutionary & environmental sciences

For a reference copy of the document with all sections, see nature.com/documents/nr-reporting-summary-flat.pdf

Life sciences study design

All studies must disclose on these points even when the disclosure is negative.

Sample size	For detection of IDH1 R132H mutation in brain tumors, we analyzed paired plasma samples and tumor tissues (n = 10). For selection of RNA markers for GBM subtyping, we studied paired plasma samples and tumor tissues from GBM patients (n = 5). For clinical analysis of GBM diagnosis and subtyping, we used plasma samples from GBM patients (n = 34) and control subjects (n = 26). The sample sizes were restricted by the availability of patient samples.
Data exclusions	No data were excluded.
Replication	All measurements were performed at least three times and all data were presented. All attempts at replication were successful.
Randomization	For clinical analysis of GBM diagnosis and subtyping, we randomized the plasma samples into two representative cohorts. In the training cohort, there are 12 disease samples and 8 control plasma samples. In the independent validation cohort, there are 22 disease and 18 control plasma samples. For other experiments, randomization was not applicable, as samples were not allocated into different experimental groups.
Blinding	All experiments were performed blinded from the clinical diagnoses. To generate the regression model, clinical diagnoses were used as outcome variables with the experimental results as predictor variables.

Reporting for specific materials, systems and methods

We require information from authors about some types of materials, experimental systems and methods used in many studies. Here, indicate whether each material, system or method listed is relevant to your study. If you are not sure if a list item applies to your research, read the appropriate section before selecting a response.

Materials & experimental systems

n/a	Involved in the study	
☐	☒	Antibodies
☐	☒	Eukaryotic cell lines
☒	☐	Palaeontology and archaeology
☒	☐	Animals and other organisms
☒	☐	Clinical data
☒	☐	Dual use research of concern

Methods

n/a	Involved in the study	
☒	☐	ChIP-seq
☒	☐	Flow cytometry
☒	☐	MRI-based neuroimaging

Antibodies

Antibodies used

IDH1 R132H (Master Diagnostica, clone H09, catalog no. MAD-000475QD7, lot 04750033, 1:1000 dilution), HSP90 (Cell Signaling, catalog no. 4874S, lot 2, 1:1000 dilution), Flotillin 1 (BD Biosciences, clone 18, catalog no. 610820, lot 4357629, 1:1000 dilution), CD63 (Santa Cruz, clone MX-49.129.5, catalog no. sc-5275, lot I2719, 1:250 dilution), ALIX (Cell Signaling, clone 3A9, catalog no. 2171S, lot 2, 1:1000 dilution), TSG101 (BD Biosciences, clone 51, catalog no. 612696, lot 5030665, 1:1000 dilution), LAMP-1 (BD Biosciences, clone 25, catalog no. bd 611042, lot 3214627, 1:1000 dilution), anti-mouse IgG, HRP-linked antibody (Cell Signaling, catalog no. 7076, lot 31, 1:2000 dilution), anti-rabbit IgG, HRP-linked antibody (Cell Signaling, catalog no. 7074, lot 28, 1:2000 dilution)

Validation

All antibodies used have been validated by the manufacturer on mammalian cells through immunohistochemistry, flow cytometry and/or western blotting.

Eukaryotic cell lines

Policy information about cell lines and Sex and Gender in Research

Cell line source(s)

GLI36vIII and SKMG3 were provided by Memorial Sloan-Kettering Cancer Center. Human glioma-propagating cell lines NNI-11, NNI-22, NNI-32 and NNI-24 were generated from primary GBM samples, provided by National Neuroscience Institute (NNI). Sex of cell lines is unavailable due to data privacy law.

Authentication

All experiments were conducted with low-passage cells for which maintenance of phenotypic, transcriptomic and karyotypic features found in the original primary tumor were previously demonstrated [Chong, Y. K. et al. Stem Cells, 27, 29-39 (2009)]. Specifically, cells maintained self-renewal and multipotentiality properties. Markers of the stemless state and differentiation markers were profiled with RT-qPCR and immunofluorescence staining. Cells were able to differentiate into neurons, astrocytes and oligodendrocytes. All cells were of tumor origin and preserved their karyotypic integrity (metaphase-fluorescent in situ hybridization and spectral karyotyping on metaphases), and maintained hallmarks of GBM (i.e., serial transplantation of glioma masses upon implantation into NOD/SCID mice).

Mycoplasma contamination

All cell lines were tested negative for mycoplasma contamination.

Commonly misidentified lines
(See ICLAC register)

No commonly misidentified cell lines were used.
